# Multiple Cell Signalling Pathways of Human Proinsulin C-Peptide in Vasculopathy Protection

**DOI:** 10.3390/ijms21020645

**Published:** 2020-01-18

**Authors:** Selma B. Souto, Joana R. Campos, Joana F. Fangueiro, Amélia M. Silva, Nicola Cicero, Massimo Lucarini, Alessandra Durazzo, Antonello Santini, Eliana B. Souto

**Affiliations:** 1 Department of Endocrinology, Hospital de São João, Alameda Prof. Hernâni Monteiro, 4200-319 Porto, Portugal; sbsouto.md@gmail.com; 2 Department of Pharmaceutical Technology, Faculty of Pharmacy, University of Coimbra (FFUC), Pólo das Ciências da Saúde, 3000-548 Coimbra, Portugal; joanacampos92@gmail.com (J.R.C.); jffangueiro@gmail.com (J.F.F.); 3 Department of Biology and Environment, University of Trás-os-Montes e Alto Douro, UTAD, Quinta de Prados, P-5001-801 Vila Real, Portugal; amsilva@utad.pt; 4 Centre for Research and Technology of Agro-Environmental and Biological Sciences, CITAB, UTAD, Quinta de Prados, P-5001-801 Vila Real, Portugal; 5 Dipartimento di Scienze biomediche, odontoiatriche e delle immagini morfologiche e funzionali, Università degli Studi di Messina, Polo Universitario Annunziata, 98168 Messina, Italy; ncicero@unime.it; 6 CREA—Research Centre for Food and Nutrition, Via Ardeatina 546, 00178 Rome, Italy; massimo.lucarini@crea.gov.it (M.L.); alessandra.durazzo@crea.gov.it (A.D.); 7 Department of Pharmacy, University of Napoli Federico II, Via D. Montesano 49, 80131 Napoli, Italy; 8 CEB—Centre of Biological Engineering, University of Minho, Campus de Gualtar, 4710-057 Braga, Portugal

**Keywords:** diabetes mellitus, C-peptide, vasculopathy, diabetic neuropathy, diabetic nephropathy

## Abstract

A major hallmark of diabetes is a constant high blood glucose level (hyperglycaemia), resulting in endothelial dysfunction. Transient or prolonged hyperglycemia can cause diabetic vasculopathy, a secondary systemic damage. C-Peptide is a product of cleavage of proinsulin by a serine protease that occurs within the pancreatic β-cells, being secreted in similar amounts as insulin. The biological activity of human C-peptide is instrumental in the prevention of diabetic neuropathy, nephropathy and other vascular complications. The main feature of type 1 diabetes mellitus is the lack of insulin and of C-peptide, but the progressive β-cell loss is also observed in later stage of type 2 diabetes mellitus. C-peptide has multifaceted effects in animals and diabetic patients due to the activation of multiple cell signalling pathways, highlighting p38 mitogen-activated protein kinase and extracellular signal–regulated kinase ½, Akt, as well as endothelial nitric oxide production. Recent works highlight the role of C-peptide in the prevention and amelioration of diabetes and also in organ-specific complications. Benefits of C-peptide in microangiopathy and vasculopathy have been shown through conservation of vascular function, and also in the prevention of endothelial cell death, microvascular permeability, neointima formation, and in vascular inflammation. Improvement of microvascular blood flow by replacing a physiological amount of C-peptide, in several tissues of diabetic animals and humans, mainly in nerve tissue, myocardium, skeletal muscle, and kidney has been described. A review of the multiple cell signalling pathways of human proinsulin C-peptide in vasculopathy protection is proposed, where the approaches to move beyond the state of the art in the development of innovative and effective therapeutic options of diabetic neuropathy and nephropathy are discussed.

## 1. Introduction

Diabetes mellitus (DM), a chronic metabolic disease affecting the homeostasis of blood glucose levels, remains an increasing health problem both in developing and developed countries [1,2,3]. This disease is associated with several complications, such as retinopathy, nephropathy, neuropathy, and cardiovascular diseases [4]. Diabetes is known by the constant high blood glucose level (i.e. hyperglycaemia), a condition that causes endothelial dysfunction. Additionally, the main biochemical pathways that influence diabetes include: the (i) polyol pathway; (ii) protein kinase C (PKC) activation; (iii) advanced glycation end-products (AGEs) pathway induced by their accumulation; (iv) activation of the hexosamine pathway; (v) oxidative stress, and (vi) expression of growth factors, such as vascular endothelial growth factors (VEGF) [5,6,7]. It is known that hyperglycemia is responsible for the development of cardiovascular diseases and premature death [8]. It is also considered as an important clinical condition of micro- and macrovascular complications in patients with diabetes (cardiovascular complications) [9,10]. Additional factors that play an important role in the setting up of type 2 diabetes mellitus (T2DM) include the chronic hyperglycemia itself but also obesity which is recognised as a risk factor for the insulin resistance development and T2DM. Many natural products of nutraceutical interest [11,12,13,14,15,16,17,18,19,20,21,22,23,24,25,26,27,28] have been proposed worldwide for the management of diabetes, as evidenced in a recent quantitative literature analysis [29]. Hyperglycemia contributes to glycosylation of proteins and lipids, enhances the production of reactive oxygen species, encourages the synthesis and secretion of pro-inflammatory cytokines and also acute phase proteins in various tissues, which promote inflammation processes in the vascular wall [30,31]. In obese patients a disturbed lipid and carbohydrate metabolism has been described, with increased inflammation and endothelial dysfunction. Janowska et al. suggest that, in obese patients with T2DM, high concentrations of C-peptide intensify inflammatory and also atherogenic processes [32]. Diabetic vasculopathy, a secondary systemic damage, is due to transient or prolonged hyperglycemia. It is known that, through the insulin or oral anti-hyperglycemic agents, it is possible to control hyperglycemia, but diabetic patients develop long-term vascular complications (even after intensive glycemic control). During anti-hyperglycemic therapy, the early or transient hyperglycemia can change cellular signalling in the vasculature. This altered microenvironment—metabolic or hyperglycemic memory (HGM)—can induce later complications, emerging thus as an underlying mechanism behind diabetic complications [33]. It was observed the pleiotropic effects on endothelial cells can be due to stimulation of insulin receptors [34]. For example, insulin enhances the endothelial uptake of amino acids and also induces vasorelaxation, and increases survival and migration of endothelial cells [35,36]. The role of insulin in physiological and pathological angiogenesis is linked to its effect in the increase of pericytes survival, reduction of anti-angiogenic protein expression, and enhancement of expression of proangiogenic factors (VEGF) [37]. Vessel formation can have multiple origins; one of them is the angiogenesis characterized by the formation of new blood vessels from pre-existing ones [38]. In vessels formation other processes are also present, including vasculogenesis (angioblast differentiate into endothelial cells leading to *de novo* vessel formation) and intussusception (split of pre-existing vessels), and vascular mimicry (tumor cell line vessels). Besides, in the revascularization of ischemic tissues other processes can also occur, such as differentiation of progenitor endothelial cells or bone-marrow-derived cells, as well as vessel differentiation into arteries (arteriogenesis). Thus, angiogenesis can impact body functions because vessels sustain almost all the body cells. Angiogenesis is thus beneficial for regeneration and tissue growth, but it is also linked to a malignant side due to the enhanced inflammatory response, development of malignant diseases and risk of cancer metastasis [39]. Expansion of new blood vessels happen in adult life through vasculogenesis, angiogenesis and arteriogenesis [34]. In certain pathologies where reduced blood vessel formation was observed, the stimuli for vessel formation can be down-regulated, as opposite to malignant diseases where up-regulation is expected. Insulin, hormone involved in tissue growth and also in recovery after injury, affect angiogenesis through some mechanisms: (i) control of the interaction between endothelium and pericytes; (ii) endothelial cell migration and proliferation (particularly in microcirculation); (iii) synthesis of pro-angiogenic factors such as VEGF and angiotensin (Ang) and (iv) regulation of tissue metabolism, which affects endothelial cell survival. These effects are well explained in the literature, but the underling signalling pathways have not yet been fully described. Particularly, in endothelial cells and pericytes functional expression of IR-A and IR-B was described, triggering intracellular signalling (PI3K and MAPK pathways) and then activation of phosphorylation cascades, producing pro-angiogenic factors (VEGF and Ang) which results in modulation of cell migration and proliferation, in vitro angiogenesis, endothelial differentiation and survival. Taking into account that angiogenesis involves these effects, insulin is considered a proangiogenic hormone, but these effects cannot be triggered in all endothelial and vascular cells [34]. Escudero et al. reviewed the information about insulin receptor activation and its intracellular pathways, highlighting the cellular outcomes and also the effect involved in insulin-mediated angiogenesis in some pathological conditions. The reduced insulin activity, in T2DM or insulin resistance, caused microvascular alterations (in skin, eye, kidney and neurovascular tissues). In diabetes, recovery of insulin effects (pharmacological use of insulin or insulin-sensitizers use) can prevent the occurrence of microvascular alterations. Nevertheless, in T2DM patients under glycemic control (in particular, when using insulin analogues), the epidemiological analyses have shown enhanced risk of developing cancer in breast, kidney, colon, prostate and pancreas [34].

## 2. Functions and Features of Human Proinsulin C-Peptide

C-peptide, present in pancreatic β-cells, is a cleavage product of proinsulin by a serine protease, being secreted in the same amount as insulin. It was regarded as a biologically inert product, being a contaminant of commercial insulin solutions [40]. However, since 1995, the biological activity of human C-peptide in diabetes has been featured given the effects in the prevention of diabetic nephropathy and neuropathy, and of vascular complications [41]. Although it is known that in diabetes many effects of vasculopathy protection are associated to the increased vascular permeability, this mechanism is not clearly understood. The main feature of diabetes, either type 1 diabetes mellitus (T1DM) and also in progressive β-cell loss in later stage of T2DM, is the lack of C-peptide [42]. In diabetes, C-peptide pathways can ameliorate endothelial dysfunction through inhibition of intracellular reactive oxygen species (ROS) mediated apoptosis of endothelial cells by activating AMP-activated protein kinase α and C-peptide can also be protective against diabetic retinopathy by amelioration of retinal microvascular leakage [43,44]. Thus, the various effects of C-peptide are attributed to the activation of multiple cell signalling pathways, involving (i) p38 mitogen-activated protein kinase, (ii) extracellular signal–regulated kinase -1/-2 (Erk-1/-2), (iii) Akt and (iv) endothelial nitric oxide (NO) production [5,6,7]. Lately, it has been shown the role of C-peptide in improving diabetes and also in organ-specific complications (microangiopathy and vasculopathy) through preservation of vascular function, as well as the prevention of endothelial cell death, vascular inflammation, microvascular permeability and neointima formation [42]. In macrovascular complications, it was suggested that C-peptide can prevent smooth muscle cell proliferation and neointima formation, being anti-atherogenic in T2DM [45,46], whereas other authors suggested a pro-atherogenic effect [47]. The use of C-peptide therapy, for the treatment of diabetic neuropathy and also nephropathy, reached clinical trials in T1DM patients [40]. It was shown that the replacement of a physiological amount of C-peptide could improve microvascular blood flow in several tissues of diabetic animals and humans (myocardium, nerve tissue, kidney and skeletal muscle) [48]. A review of the multiple cell signalling pathways of human proinsulin C-peptide in vasculopathy protection is here proposed, discussing the approaches to move beyond the state of the art in the development of innovative and effective therapeutic options of diabetic neuropathy and nephropathy.

## 3. Cell Signalling Pathways of Human Proinsulin C-Peptide in Vasculopathy Protection

**Figure 1:**
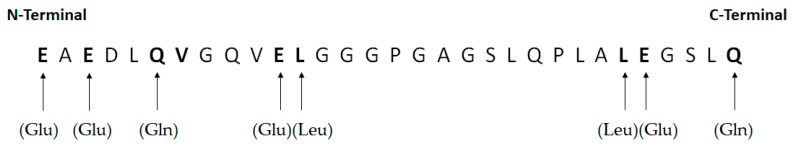
Amino acid sequence of human C-peptide: E (glutamic acid, Glu), A (alanine), D (aspartic acid) L (leucine, Leu), Q (glutamine, Gln), V (valine), G (glycine), P (proline) and S (serine) (Adapted from [49]).

Human C-peptide (C_129_H_211_N_35_O_48_) has 31-amino acids cleaved off from the proinsulin molecule in the course of insulin biosynthesis, and is stored in secretory granules in the pancreatic beta cells. It has an important role in the synthesis of insulin, i.e., serving as a linker between the insulin A- and B-chains, allowing the formation of the interchain disulfide bridges in the insulin molecule [49]. It is considered an acidic peptide, because it has five acidic residues in its human form (up to seven in other species) without any basic residues. There is an abundant inter-species variability concerning the amino acid sequence and the number of residues, and the variation in size occurs in the molecule’s central region. Other species (e.g., mouse and rat) have two isoforms of C-peptide and proinsulin that are also different. In mammals, amino acid sequence for human C-peptide has eight well conserved residues (bold in Figure 1) [49].

Through its structure, it is possible to see that the acidic residues (especially Glu) are important for C-peptide bioactivity. Glu 27 is important in direct measurement of cellular responses to distinct analogues [50,51]. As mentioned above, it was thought that C-peptide was biologically inert, until it was proven that it activates intracellular signalling pathways in various cell types [4]. C-peptide, physiologically active, ameliorates the complications induced by diabetes. It is proven that hyperglycaemia, deficiency or absence of circulating insulin and/or C-peptide can affect body homoeostasis interfering in the development and progression of complications induced by hyperglycaemia. Studies using various models of diabetes indicate that in vivo hyperglycaemia induces VEGF expression and production of ROS in endothelial cells of diabetic retinas, but the molecular mechanisms of induction of hyperglycemia are not yet fully understood [42]. C-peptide can inhibit mitochondrial dysfunction, transglutaminase 2 activation, and endothelial cell apoptosis, among other intracellular events mediated by ROS [33]. In nanomolar concentrations, C-peptide connects with G-protein coupled receptor in cell membranes. Signalling pathways dependent of Ca^2+^ and MAP-kinase are activated, stimulating Na^+^, K^+^-ATPase and endothelial NO synthase (deficient in diabetes). C-peptide also interacts with insulin signal transduction in a synergistic way. In T1DM, it enhances blood flow in various tissues via stimulation of endothelial NO release, demonstrating the action of C-peptide in keeping vascular homeostasis [49]. Through hypoglycemic, antioxidant, antiapoptotic, and anti-inflammatory effects, C-peptide has also a protective effect against atherosclerosis. Thus, it can help as a therapy in late stages of T2DM by decreasing incidence of vasculopathy. Rheim et al. investigated the relationship between C-peptide and atherosclerosis in adult male albino rats in late stages of experimentally induced T2DM, proving that administration of C-peptide can decrease complications of endothelial dysfunction and also reduce insulin dose to prevent hyperinsulinemia [4].

In macrovascular complications, it is suggested that C-peptide avoids smooth muscle cell proliferation and neointima formation, being anti-atherogenic but other studies showed a pro-atherogenic effect [45,52,53,54]. In some tissues in diabetic animals and patients, the replacement of an amount of C-peptide has shown to improve microvascular blood flow [48,55]. C-peptide prevents endothelial dysfunction in vasculature through activation of Erk-1/-2 and endothelial NO synthase, production of NO or inhibition of nuclear factor kappa light-chain enhancer of activated β cells pathway [56,57]. Lim et al. studied how C-peptide ameliorates endothelial dysfunction through the inhibition of intracellular ROS-mediated apoptosis of endothelial cells, but the way in which it contributes with beneficial effects on diabetic vascular complications is not clearly understood [42].

Diabetes is also characterized by an increase of VEGF expression in retina, with the risk of promoting neovascularization and macular oedema [58,59,60]. The increase of VEGF is associated to the generation of ROS in retinal endothelial cells, which contributes for vascular complications [61,62]. VEGF is known to be a mediator of vascular alteration in diabetic retinopathy, and its expression is increased by high glucose levels, hypoxia, oxidative stress and by inflammatory reactions. VEGF is a powerful promoter of physiological angiogenesis [58,59,60]. In diabetes, increased vascular permeability is stimulated by the VEGF overexpression, which breaks adherens junction proteins (VE-cadherin) caused by the elevation of intracellular Ca^2+^ and ROS levels [63,64,65,66].

Lim et al. studied the physiological functions of C-peptide in microvascular permeability induced by hyperglycemia, using human umbilical vein endothelial cells (HUVECs) and streptozotocin diabetic mice. The authors showed that C-peptide has a protective effect on microvascular permeability induced by VEGF through the intracellular ROS generation, stress fibre formation and adherens junction integrity in endothelial cells. In the peripheral vessels of the skin of diabetic mouse, C-peptide was shown to prevent vascular permeability. C-peptide also induced the prevention of microvascular leakage in the retinas of streptozotocin diabetic mice, anti-VEGF antibody and ROS scavenger effect, which led to the conclusion of the potential use of C-peptide replacement therapy to prevent microvascular permeability of the retina induced by diabetes and retinopathy. This study demonstrated elevation of intracellular Ca^2+^ and ROS and formation of stress fibres induced by VEGF. The generation of intracellular ROS induced by VEGF was shown to be prevented by C-peptide and to be unleashed by the elevation of intracellular Ca^2+^ [42]. The role of C-peptide in preventing elevated ROS generation induced by glucose without interfering with intracellular Ca^2+^ levels has been demonstrated in previous studies using endothelial cells [43]. It has also been demonstrated that C-peptide does not have inhibitory effect on the elevation of intracellular Ca^2+^ levels induced by VEGF, which corroborates the conclusion that C-peptide plays a role in the prevention of vasculopathy. C-peptide inhibits ROS generation induced by VEGF by a mechanism involving AMP-activated protein kinase a (AMPKa) [42]. It was proven that C-peptide activated AMPKa, preventing hyperglycaemia induced by ROS generation and thus cell damage [67]. VEGF increases ROS generation dependent on NADPH oxidase, being inhibited through AMPKa activation by C-peptide [68]. Stress fibre formation in some permeability-increasing mediators (thrombin and TNF-α) allows the increase of permeability [65]. VEGF (ROS generation’s mediator)-induced stress fibre formation, being reduced by C-peptide and ROS scavengers Trolox and NAC. C-peptide also prevented the disassembly of VE-cadherin induced by VEGF (an endothelial cell specific adhesion molecule responsible for connecting adjacent endothelial cells). Phosphorylation of VE-cadherin and β-catenin is determined by ROS generation and stress fibre formation in decreasing junctional integrity and in increasing vascular permeability [64]. It has thus been shown that VEGF can dissimulate VE-cadherin at adherens junctions, while C-peptide avoids disassembly induced by VEGF of VE-cadherin. This inhibition of disassembly induced by VEGF of VE-cadherin by NAC and Trolox has denoted that ROS inhibition by C-peptide is crucial to defend against disassembly induced by VEGF of VE-cadherin. Therefore, through inhibition of disassembly of adherens junction mechanisms involved in intracellular ROS generation and stress fibre formation, C-peptide prevents vascular permeability induced by VEGF in endothelial cells. Besides, VEGF can also produce NO in endothelial cells [42,69]. Elevation of ROS and NO induced by VEGF originates peroxynitrite flux, improving vascular permeability [70]. Nevertheless, C-peptide inhibition of ROS generation induced by VEGF can impair the formation of peroxynitrite also induced by VEGF. Thus, C-peptide inhibition of ROS generation also impairs vascular permeability induced by VEGF through the inhibition of peroxynitrite production. C-peptide may therefore protect against microvascular permeability induced by VEGF as evidenced in the skin and retina of streptozotocin diabetic mice. In late stages of diabetes, C-peptide offers protection against harmful effects of VEGF-induced microvascular permeability, preventing the development and progression of macular oedema and the progression of proliferative angiogenesis. This is similar to the role of angiopoietin-1 that also protects against vascular permeability by inhibiting VE-cadherin disruption [71,72]. Nevertheless, the protective effect of C-peptide of vascular permeability is governed by restriction of ROS production, and thus restriction of stress fibre formation and consequent disruption of VE-cadherin at the endothelial junctions, but ROS are not associated with the mechanism of action of angiopoietin-1 [73]. Therefore, C-peptide was shown to exhibit positive effects against destructive outcomes by signalling mechanisms different from those of angiopoietin-1. As C-peptide inhibits ROS generation induced by hyperglycaemia, it also shows an anti-apoptotic effect in endothelial cells [43,74,75]. Thus, the absence of C-peptide provides appropriate physiological environment for the development and progression of diabetic complications through the increase of pathological stimuli (ROS generation, apoptosis and vascular permeability). The beneficial effects of C-peptide against pathologies induced by diabetes, may be an advantages in replacement therapies together with insulin treatment for both types of diabetes [42].

C-reactive protein (CRP), acute phase protein, is produced in the liver through pro-inflammatory adipocytokines tumor necrosis factor alpha (TNFα) and interleukin-6 (IL-6), known to be a recognized inflammation marker. Besides, in atherosclerotic plaque its local source are the macrophages, being also involved in the formation and progression of atherogenic lesions. In macrophages, it has the potential to promote secretion of tissue factor, inflammatory cytokines and also ROS, induces monocyte chemotaxis and adhesion, as well as the formation of oxidized low-density lipoprotein (oxLDL) [76]. In the smooth muscle cells, it has the potential to increase the production of inducible nitric oxide synthase (iNOS) and activity of nuclear factor kappa-light-chain-enhancer of activated B cells (NF-κβ), as well as mitogen-activated protein kinases (MAPK) pathways. This protein encourages receptor’s expression for angiotensin, allows the raise in the synthesis of free radicals, migration and proliferation of smooth muscle cells, activation of complement pathway and also tissue factor expression, and lipids uptake by macrophages [77]. And in patients with T2DM, is an indication of chronic kidney disease [78]. The increased levels of C-peptide are also correlated with excessive secretion of insulin, secondary to insulin resistance. Through specific G-protein-coupled receptor, C-peptide connects to cell membranes, activating different signalling pathways (kinases pathway associated with MAP and Erk-1/-2 proteins, protein kinase C and also phosphoinositide 3-kinase, as well as stimulation of Na^+^ and K^+^-ATPase) and also stimulates angiogenesis processes by an activation of extracellular signal-related kinase -1/-2 and AKT phosphorylation and NO synthesis [44,46,79]. In a study related to the development of late complications in T1DM, the neuronal nephroprotective activity of C-peptide has been highlighted. The supplementation with C-peptide in T1DM exclusively has also shown to ameliorate microvascular complications [80].

The factors that affect the development of the atherogenic process include C-peptide, which has a special role. In T1DM, the deficiency in this peptide affects microvascular complications, but its increase causes its deposition in the intima of vessels in early stages of T2DM and also in patients that have insulin resistance. These deposits are the cause of infiltration of inflammatory cells and proliferation of smooth muscle cells. Janowska et al. studied the association of fasting C-peptide concentrations and interleukin-10 (IL-10) as well as E-selectin and triglycerides levels, showing that C-peptide influences the diabetic atherogenesis [32]. Lee et al. demonstrated that IL-10 and the diabetic retinopathy in T2DM patients are inversely proportional [81]. It is also known that IL-10 affects endothelial function by stimulating different signalling pathways. IL-10 exhibits anti-inflammatory activity through the restriction of the activation of inflammatory cytokines genes transcription (transcription factor NF-κβ), initiation of suppressor of cytokine synthesis-3 and stimulation of Th2 cytokines, as well as in defense of vascular dysfunction induced by angiotensin II (Ang II) [82]. Moreover, in T2DM patients it was noted an association, suggesting that IL-10 can regulate the glucose metabolism being negatively correlated with HOMA-IR. These conclusions are in line with results obtained with transgenic mice in which an overexpression of IL-10 was observed, preventing muscle insulin resistance induced by diet [83]. Therefore, Janowska et al. suggest that elevated concentrations of C-peptide intensify the inflammatory and atherogenic processes in obese T2DM patients [32].

As an underlying mechanism of diabetic complications is the HGM [84]. Hyperglycemia contribute to extreme generation of mitochondrial ROS, which in turn activate the PKC and NADPH oxidase causing cytosolic ROS generation [33]. HGM also persists because of epigenetic modifications (hypermethylation of transcription-activating histone H3 residue Lys4, de-methylation of transcription-repressive histone H3 residue Lys9 and acetylation of histone H3) [85,86]. These modifications result in the up-regulation of transcription factor p53 as well as mitochondrial adaptor protein p66shc, which are responsible for sustained ROS generation, endothelial damage or delayed wound healing in diabetes [87,88,89]. The ROS production is crucial in the initiation of pathways that are responsible for the pathogenesis of diabetic vasculopathy (polyol and hexosamine pathway flux, advanced glycation end-product formation and activation of protein kinase C) [90,91,92]. Besides, superoxide generation induced by hyperglycemia and eNOS uncoupling result in the cellular nitrosative stress (due to peroxynitrite (ONOO^−^) and nitrotyrosine formation), contributing to the damage of endothelial cell [93,94]. It is known that NO production dependent of eNOS stimulates vasodilation, but the overproduction of O2^−^ withdraw the accessible NO, enhancing the development of intracellular ONOO^−^ as well as nitrotyrosine in diabetic vasculopathy [95,96]. with the correlation between HGM and cardiovascular complications in DM is very well known; thus in order to prevent long-term cardiovascular complications associated with diabetes, it is recommended the premature aggressive treatment of glycemic control as well as the supplementation with agents which defend against the generation of intracellular reactive oxygen and nitrogen species [84,97,98]. In this context, Lim et al. used the retinas of diabetic mice to show that C-peptide has a protective effect in microvascular permeability induced by vascular endothelial growth factor and they also show that the stimulation of angiogenesis and constriction of inflammation reduce impaired wound healing by C-peptide replacement therapy [42,44]. The therapeutic management of hyperglycemia is known to be instrumental to avoid diabetic complications, and this is achieved by regular insulin administration in T1DM, the use of oral hypoglycemic agents in the early stages of T2DM as well as the use of insulin and also hypoglycemic agents in the later stages of T2DM [99,100]. However, it has not possible yet the prevention or control of diabetic complications through the use of intensive insulin therapy [86,97]. ROS generation induced by chronic or transient hyperglycemia is crucial in the pathogenesis of diabetic vasculopathy (nitrosative stress and endothelial apoptosis) [99]. Even after the maintenance of normoglycemia, these processes have not been totally controlled and additional antioxidant therapies have shown limited beneficial results [101]. As C-peptide has the capacity to inhibit adverse cellular events related to HGM by reducing ROS, ONOO^−^ as well as nitrotyrosine formation, Bhatt et al. confirmed that endogenous C-peptide can be used as a first-line therapeutic supplement in diabetic patients [33]. Previous studies have provided insights about the molecular mechanisms involved in the progression induced by HGM diabetic vascular complications despite glycemic control, demonstrating that continuous ROS production promotes HGM-induced complications [87,89,92]. As continuous ROS overproduction induced by hyperglycemia is a crucial step in the induction of endothelial apoptosis in diabetic vasculopathy, this overproduction after glucose stabilization in human endothelial cells and in aortas has been investigated in diabetic mice [33]. Bhatt et al. studied the role of C-peptide in the apoptosis of vascular endothelial cells induced by HGM and observed that C-peptide inhibited p53 upregulation and p66shc activation and ROS generation, as well as nitrosative stress and thus endothelial apoptosis in human endothelial cells and in the murine aortas. These studies demonstrate that C-peptide can be a good therapeutic candidate to treat diabetic vascular damage induced by HGM [33].

Studies suggest that some processes (like β-cell calcium dyshomeostasis, changed protein folding and also oxidative stress) start early in the progression of T1DM, and can increase β-cell death mediated by autoimmune through the triggering of β-cell endoplasmic reticulum (ER) dysfunction or ER stress [102]. The β-cell–secretory endocrine cell–has a robust and functional ER in order to provide the required production and folding of proteins (insulin). Under conditions that impair ER health, insulin demand can compromise the competence of the ER to process recently translated proteins (ER stress). Unchecked ER stress can lead to β-cell death, but the non-invasive identification of this process provides the monitoring of the evolution of disease and allows recognition of individuals who have the risk to develop T1DM before the β-cell destruction. The β-cell ER dysfunction is known to accumulate and secrete inadequately processed proinsulin (PI) molecules [103]. Thus, it is possible to detect in a non-invasive way the β-cell ER stresses, measuring the serum PI-to-serum C-peptide (PI:C) ratio that is released in a 1:1 molar ratio with mature and entirely processed insulin [104,105]. It was previously recognised that the β-cell ER dysfunction is characteristic of the autoimmune process. Sims et al. have already demonstrated an increase in the relation of PI:C as function of time of clinical onset of T1DM in murine and in human models. They hypothesized that an increase in the PI:C ratio also exist in subjects with high risk (even before the start of meaning hyperglycemia), serving to prognosticate T1DM development. Therefore, their findings confirm the relevance of β-cell ER dysfunction as a contributor to the development of T1DM in humans. The use of monitoring of the PI:C ratio as signal of β-cell stress can be useful as a surrogate or intermediate outcome measure in treatment testings, allowing to escalate or change therapy [106]. A recent study reported an association of small pro-insulin C-peptide with the small lumbar mineral density in postmenopausal woman without diabetes, but a specific role of C-peptide on bone biology has never been described [107]. Russo et al. clarified how C-peptide can activate and proliferate the intracellular pathways of human osteoblast-like cells (Saos-2), providing additional information about the expression of the bone extracellular matrix proteins. These authors have shown, for the first time, that C-peptide activates a specific intracellular pathway in osteoblasts and also that, together with insulin, it can influence collagen biosynthesis and RANKL expression [108].

## 4. Development of Innovative and Effective Therapeutic Options in Diabetic Neuropathy

In patients with diabetes it is possible to find several types of disturbances that influence the peripheral and autonomic nervous system. Distal symmetric sensory polyneuropathy is the commonest type of neuropathy, involving small fibre sensory modalities early in the course of the disorder. There is still no therapy for diabetic neuropathy, although many compounds have already been tested. Most of them are aldose reductase inhibitors as well as agents that inhibit the formation of advanced glycation end-products, with the aim to relief of the metabolic outcomes of hyperglycemia. However, they have limited efficacy and present adverse reactions. Studies made in animal models and in patients with T1DM have shown that C-peptide in replacement doses can enhance peripheral nerve function and prevent/reverse the development of nerve structural changes [49]. The replacement of C-peptide in diabetes in BB/Wor rats, an established model of T1DM, has shown to counter the deterioration of the nerve conduction velocity (NCV, sciatic–tibial nerve) in comparison to non-replaced diabetic control animals. The administration of this peptide decreases the NCV defect, exhibiting a beneficial effect on the structural changes. It also allows the prevention of diabetes-induced paranodal swelling, axoglial disjunction as well as paranodal demyelination of sural nerve fibres. The use of C-peptide treatment after diabetes, when neuropathy has been established, prove to improve the NCV and to marked repair of diabetes-induced axonal degeneration and increased nerve fibre regeneration (sural nerve) [109]. The above-mentioned effects on nerve function and structure in the diabetic rat can be elicited also by human C-peptide at higher concentrations [110]. Increasing doses of human C-peptide results in lower marked early structural abnormalities and a growing frequency of regenerating sural nerve fibres, which demonstrated cross-species activity of human C-peptide and dose dependency of the C-peptide effect [111]. Diabetes causes reduction in motor (sciatic) and sensory (saphenous) nerve conduction velocities. C-peptide administration at physiological concentrations prevented the motor and sensory NCV deficit. Neurovascular mechanisms are relevant for the C-peptide effect on nerve function, which was proven when the co-administration of C-peptide and an eNOS blocker completely abolished the C-peptide effects on NCV [112]. It has also been demonstrated the positive effects of C-peptide on nerve function in patients. In a study, T1DM patients without symptoms of neuropathy and with diabetes were given C-peptide or placebo together with their regular insulin regimen. At the beginning of the study, sensory (sural) and motor (peroneal) nerve conduction velocities were reduced in comparison to the healthy controls. A significant increase in sensory NCV was recorded after the administration of C-peptide, which represented a correction of the initial defect. Vibration perception thresholds improved, but the motor NCV remained unchanged [113]. Similar findings have been found in other group of patients, where lowered thermal perception thresholds were observed after C-peptide replacement, which suggests enhanced sensory nerve function [114]. Therefore, these data prove that a therapeutic improvement of diabetes-induced peripheral nerve dysfunction can be seen after C-peptide administration in patients with T1DM [49]. The effects of this peptide have also been evaluated in T1DM patients with early signs of autonomic dysfunction. The patients were studied under normoglycemic conditions and during an intravenous infusion of either human C-peptide or saline, where plasma concentrations of C-peptide during the infusion reached levels within the physiological range. Heart rate variability during deep breathing (an index of autonomic nerve activity—primarily vagal) was decreased before the study and increased during C-peptide but not during saline infusion [115]. Besides, an improvement in the brake index during tilting was found in the patients who had a reduced index before the study. During saline infusion, there is no response. In another study, autonomic nerve function has also been evaluated in T1DM receiving C-peptide replacement [114]. An improvement in heart rate variability was found in C-peptide administration, while no change or deterioration was observed in the same patients in the control period. The stimulatory influence of C-peptide on the parasympathetic system can be an effect on the vagal nerves, but a direct action on the central nervous system has also been proposed [116]. The pathogenic mechanisms involved in the development of distal symmetric sensory polyneuropathy have several causes: metabolic effects mediated by hyperglycemia, hypoxia due to by microvascular disease as well as the impaired neurotrophic support, or just a combination of these causes. It is known that strict metabolic control will retard (but not prevent) the development of diabetic neuropathy, indicating that other factors besides hyperglycemia are also important [49]. The impaired nerve blood flow secondary to perturbed NO metabolism and reduced levels of nerve Na^+^, K^+^-ATPase activity are implicated in the pathogenesis of distal symmetric sensory polyneuropathy [110,117,118]. Therefore, C-peptide can partially correct the reduction of Na^+^, K^+^-ATPase activity that is associated with experimental diabetes [109,110]. Moreover, in vitro studies suggest that C-peptide with insulin stimulates neuroblastoma cell proliferation and neurite outgrowth and decreases glucose induced apoptosis. It also enhances the expression and translocation of nuclear factor jβ (NF-jβ) and promotes Bcl2 expression in neuroblastoma cells, which can contribute to the anti-apoptotic effects of C-peptide [49,119]. NF-jβ contributes to nerve cell plasticity, development and differentiation, being in agreement with the effect of C-peptide on nerve function and structure in diabetes [49].

## 5. Development of Innovative and Effective Therapeutic Options in Diabetic Nephropathy

Diabetic nephropathy, one of the microvascular diabetic complications, is clinically characterized as the progressive development of renal insufficiency with hyperglycemia. This disease is the major single cause of end-stage renal failure in several countries [120]. Early signs of diabetic nephropathy include renal hypertrophy, glomerular enlargement due to mesangial matrix expansion and glomerular hyperfiltration and loss of renal functional reserve [49]. There are some theories for the pathogenic mechanisms which result in the development of diabetes-induced renal dysfunction. Early in the course of T1DM, specific organs’ function deteriorates and tissue abnormalities arise and hyperglycemia results in abnormal homeostasis in blood flow and vascular permeability in the glomerulus. The increased blood flow and intra-capillary pressure reveal hyperglycemia-induced decreased NO production on the efferent side of renal capillaries and an increased sensitivity to Ang II. Consequently, glomerular capillaries have a higher albumin excretion rate. In this early stage, the increased permeability is still reversible, but below the continuing triggering effect of hyperglycemia the lesions become irreversible [121]. Besides the two major factors, insulin deficiency and subsequent hyperglycemia, which contribute to the development of diabetic complications, C-peptide deficiency is suggested to be the third major factor because of beneficial effects of C-peptide against diabetic complications [40]. Luppi and Drain reported that C-peptide has an anti-oxidant effect on the -cells producing it, which limit cell dysfunction and loss contributing to diabetes and suggesting a positive feedback that might potentiate the endogenous and exogenous C-peptide benefits on the kidney [122]. Renal sympathetic nervous system, an important regulator of insulin resistance, shows that renal nerve ablation substantially improves insulin sensitivity and glucose metabolism, in addition to significantly reducing blood pressure. Percutaneous renal denervation can represent the first non-pharmaceutical approach for treating insulin resistance and drug-resistant hypertension. Activation of the sympathetic nervous systems contributes to insulin resistance and the metabolic syndrome and it is associated with central obesity and risk of developing DM [123,124,125,126]. Although insulin itself exhibits sympatho-excitatory effects, renal denervation allows examination of the direct role of the sympathetic nervous system, without causing further systemic pharmacological interactions, in mediating insulin resistance and its consequences [127,128,129]. The influence of C-peptide administration on the renal function and structure in diabetic nephropathy has been examined both in animal models of diabetes and in T1DM patients, especially in the early stages of the disorder [49]. Elbassuoni et al. studied the C-peptide replacement therapy on diabetic nephropathy and its mechanisms of action, by understanding the function of NO as a mediator of C-peptide effects by in vivo modulating its production by NG-nitro-L-arginine methyl ester (L-NAME). The measured renal injury markers were serum urea, creatinine, tumor necrosis factor alpha, and Ang II, and malondialdehyde, total antioxidant, Bcl-2 and NO in renal tissue. Diabetic induction caused islet degenerations and decreased insulin secretion with its metabolic consequences and subsequent renal complications. C-Peptide deficiencies in diabetes might have contributed to the metabolic and renal error, since C-peptide treatment to the diabetic rats completely corrected these errors. The beneficial effects of C-peptide are partially antagonized by L-NAME coadministration, which indicated that NO partially mediates C-peptide effects [130]. Renal sympathetic afferent and efferent nerves play an important role in blood pressure regulation. Enhanced renal sympathetic drive is a common feature in patients with various forms of hypertension and it is associated with components of the metabolic syndrome [123,131,132]. There is a bidirectional relationship between sympathetic over activity which induced insulin resistance and hyperinsulinemia producing sympathetic activation and thus initiating a vicious cycle [133]. Given the involvement of the sympathetic nervous system in metabolic control, it is speculated that reduction of sympathetic activity by renal denervation can have a substantial effect on glucose metabolism in hypertensive patients. Mahfoud et al. evaluated the relationship between sympathetic activity and glucose metabolism and the role of therapeutic renal denervation in patients with resistant hypertension. They investigated the effect of renal sympathetic denervation based on catheter on glucose metabolism and blood pressure control in patients with resistant hypertension. Renal denervation enhances glucose metabolism and insulin sensitivity in addition to a significantly reduction in the blood pressure. However, this improvement appeared to be unrelated to changes in drug treatment [129]. Elbassuoni et al. described the role of C-peptide replacement therapy in diabetic nephropathy as one of the most serious diabetic complications, and its potential mechanisms of action. They planned to: (i) induce experimental T1DM by streptozotocin (STZ) in adult male albino rats, (ii) study the effects of C-peptide treatment in the developed renal injury and (iii) study the role of NO as a mediator of C-peptide effects by modulating its production in vivo by blocking its synthesis using the nitric oxide synthase (NOS) inhibitor: L-NAME. They observed that STZ injection to rats induced diabetic pictured similar to T1DM in humans, which resulted in islet degenerations and decreased insulin secretion with its metabolic consequences and subsequent renal complications. C-Peptide deficiencies in diabetes can contributed to the metabolic and renal error, as C-peptide treatment to the diabetic rats corrected completely these errors. The beneficial effects of C-peptide were partially antagonized by L-NAME coadministration, indicating that NO partially mediates the effects of C-peptide [130]. The physiological mechanisms underlying the beneficial effects of C-peptide on renal function and structure in diabetes are not completely understood. Stimulation of eNOS in renal capillary endothelial cells can modify the intraglomerular blood flow and pressure distribution, which resulted in a reduced transglomerular driving force and decreased albumin excretion. Improved renal function can also be a consequence of stimulation by C-peptide of glomerular or tubular Na^+^, K^+^-ATPase activity. C-peptide, via its activation of the MAPK-complex, can promote growth and survival of tubular cells and interrupt or interfere with the intricate pattern of growth factor signalling in glomerular tissues, thereby limiting glomerular expansion [49].

## 6. Conclusions

Since the discovery of C-peptide about 40 years ago, it received little attention as a potentially bioactive peptide. Nonetheless, during the past decade, many studies have shown new and compelling evidences that C-peptide is a biologically active peptide with multifaceted effects. It has even been proven that C-peptide deficiency is a crucial factor in the pathogenesis of long-term complications of T1DM. Since C-peptide replacement as well as insulin administration in these patients offers hope for a better therapeutic, it is being currently evaluated in clinical trials. C-peptide can protect microvascular leakage in diabetic mice, preventing VEGF-induced microvascular permeability as was shown through the inhibition ROS-mediated intracellular events, stress fiber formation, and also VE-cadherin disruption, as well as endothelial permeability in diabetic mice. Selective denervation of the renal sympathetic nerves, in the absence of significant changes in body weight as well as alterations in lifestyle or antihypertensive medication, can improve glucose metabolism and also blood pressure control in patients with resistant hypertension. Therefore, sympathetic activation is underlying the origin of linked disorders of hypertension as well as metabolic syndrome cluster of pathologic health conditions, proving that both crucial features of cardiovascular risk can be approached simultaneously. Alternative therapeutic approaches to hyperglycemic control are necessary to target hyperglycemia and diabetic complications. In this field, several clinical and experimental studies demonstrated that C-peptide treatment, alone and/or also in combination with insulin, has aphysiological functions, being beneficial in the prevention diabetic complications; studies on follow up, use, and compliance, as reported by recent publications in the area [134,135,136,137] and communication strategies and assessment [138] are nevertheless still needed. A summary of the cytoprotective, antiapoptotic and anti-inflammatory effects of C-peptide, on the renal function and on diabetic neuropathy is provided in Table 1.

## Figures and Tables

**Table 1:** Effects of C-peptide on cytoprotecting (against cell apoptosis and inflammation), on the renal function and on diabetic neuropathy (adapted from [40]).

**Cytoprotective, Antiapoptotic and Anti-Inflammatory Effects of C-Peptide**	↑ 5rc-kinase ↑ Pl-3 kinase ↑Erk-1/-2	↑ VSMC proliferation		↑ vascular damage
	↑Pl-3	↑ Chemotaxis of leukocytes		
	↓ ROS ↓ NF-kβ	↓ proliferation and migration of VSMC		↓ Vascular damage
		↓ IL-8 ↓ MCP-1	↓ leukocyte adhesion and migration into the vascular wall	
		↓ P-selectin ↓ ICAM-1 ↓ VCAM-1	↓ Leukocyte endothelium interaction	
		↑ Bcl-2 ↓ Caspase-3		↓ Apoptosis
**Effects of C-Peptide on the Renal Function**	NF-kβ PPAR_Υ_	Transcriptional effects	↑ IGF-1 ↓ TNFα ↓ TGFβ	Improved renal structure ↓ Apoptosis ↓ Mesangial expansion
	↓ Na^+^ ATPase ↓ K^+^ ATPase	↓ Na^+^ excretion ↑ Energy status		Improved renal structure Improved glomerular function ↓ Apoptosis ↓ Mesangial expansion ↓ Glomerular hyperfiltration ↓ Albumin excretion ↑ RBC deformability
	↓ eNOS	↑ Endothelial function		Improved glomerular function ↓ Glomerular hyperfiltration ↓ Albumin excretion ↑ RBC deformability
**Effects of C-Peptide on Diabetic Neuropathy**	c-fos c-jun	↑ Neurotropic factors	↑ NGF ↑ IGF-1 ↑ NT3	Improved nerve structures
	↑ eNOS	↑ Na^+^ ATPase ↑ K^+^ ATPase ↑ Endothelial function ↑ Nerve blood flow	Improved nerve function	
	↑ Na^+^ ATPase ↑ K^+^ ATPase	↓ Na^+^ retention ↑ Energy status		

## Abbreviations

AGEs	Advanced glycation end-products
AMPKa	AMP-activated protein kinase a
Ang	Angiotensin
Ang II	Angiotensin II
CRP	C-reactive protein
DM	Diabetes mellitus
ER	Endoplasmic reticulum
Erk-1/-2	Extracellular signal–regulated kinase -1/-2
HGM	Hyperglycemic memory
HUVECs	Human umbilical vein endothelial cells
IL-10	Interleukin-10
IL-6	Interleukin-6
iNOS	Inducible nitric oxide synthase
L-NAME	NG-nitro-L-arginine methyl ester
MAPK	Mitogen-activated protein kinases
NCV	Nerve conduction velocity
NF-jβ	Translocation of nuclear factor jβ
NF-κβ	Nuclear factor kappa-light-chain-enhancer of activated B cells
NO	Nitric oxide
NOS	Nitric oxide synthase
ONOO^−^	Peroxynitrite
oxLDL	Oxidized low-density lipoprotein
PI	Proinsulin
PI:C	PI-to-serum C-peptide
PKC	Protein kinase C
ROS	Reactive oxygen species
Saos-2	Human Osteoblast-like cells
STZ	Streptozotocin
T1DM	Type 1 diabetes mellitus
T2DM	Type 2 diabetes mellitus
TNFα	Tumor necrosis factor alpha
VEGF	Vascular endothelial growth factor
